# Periodontitis and pancreatic cancer: the oral–gut microbial axis, pathogenic mechanisms, and potential therapeutic implications

**DOI:** 10.1080/20002297.2026.2758491

**Published:** 2026-11-12

**Authors:** Liuqian Fan, Gaoying Hong, Jingqing Sun, Yanyan Zhou, Jue Shi

**Affiliations:** a Stomatology Hospital, School of Stomatology, Zhejiang Provincial Clinical Research Center for Oral Diseases, Zhejiang Key Laboratory of Oral Biomedical, Zhejiang University School of Medicine, Zhejiang-Singapore International Joint Laboratory of Oral Bioengineering, Hangzhou, China

**Keywords:** Periodontitis, pancreatic cancer, oral microbiota, pathogenesis, treatment strategy

## Abstract

**Background:**

Pancreatic cancer is a highly aggressive malignancy with a poor prognosis. Epidemiological studies have suggested a possible association between periodontitis and pancreatic cancer, while microbiome studies have reported alterations in the oral, gut, and pancreatic microbiota.

**Objective:**

This review examines the evidence linking periodontitis to pancreatic cancer, with particular attention to microbial interactions along the proposed oral–gut microbial axis.

**Design:**

This narrative review synthesizes current epidemiological, microbiome, and experimental evidence relevant to the potential association between periodontitis and pancreatic cancer.

**Resluts:**

Experimental studies suggest that periodontitis-associated microorganisms, including *Porphyromonas gingivalis* and *Fusobacterium nucleatum*, or their products, may reach the pancreas via digestive or circulatory routes. These microorganisms may influence the development and progression of pancreatic ductal adenocarcinoma through chronic inflammation, immune evasion, virulence factors, microbial metabolites, and the activation of oncogenic signaling pathways. Specific oral and gut microbial features have been investigated as candidate biomarkers. Periodontal treatment, dietary modification, and probiotics have also been studied for their effects on microbial composition and inflammation-related pathways potentially relevant to pancreatic cancer. The clinical relevance of these candidate biomarkers remains to be established, and microbial alterations may either contribute to pancreatic cancer or occur as a consequence of the disease.

**Conclusions:**

Current evidence supports a possible link between periodontitis and pancreatic cancer, although the role of microbiota in this relationship remains under investigation.

## Introduction

Pancreatic cancer remains one of the most lethal malignancies and comprises several histological subtypes, of which pancreatic ductal adenocarcinoma (PDAC) is the predominant type. Recent data from the Surveillance, Epidemiology, and End Results (SEER) Program and American Cancer Society (ACS) indicate that the 5-year relative survival rate of pancreatic cancer ranges from 13% to 14%, and the 2026 SEER update records an exact figure of 13.7% [1,2]. Global estimates indicate that pancreatic cancer cases have continued to rise, increasing from 195,000 in 1990 to 448,000 in 2017 and reaching 510,992 worldwide in 2022 [3,4]. The established risk profile of pancreatic cancer comprises three principal categories: demographic characteristics such as advanced age and gender; modifiable lifestyle factors, including tobacco use, alcohol consumption, dietary patterns, and physical activity levels; and pathological and metabolic conditions such as chronic pancreatitis, obesity, and type 2 diabetes [5,6]. Associations have also been reported between pancreatic cancer and systemic disorders, including inflammatory bowel disease and peptic ulcer disease [7,8]. This multidimensional risk framework underscores the complex etiology of pancreatic cancer, with increasing scientific attention directed toward oral health parameters as potential contributing factors.

Recent studies suggest a possible association between periodontitis and pancreatic cancer. Epidemiological surveys report that smoking and alcohol consumption are more common among individuals with periodontal disease; both are recognized behavioral risk factors for pancreatic cancer [9–11]. After adjusting for age, smoking history, diabetes, and other potential confounders, participants who reported a history of periodontal disease had a 64% higher risk of pancreatic cancer than those who did not [12]. Microbiological investigations suggest that oral microbiota dysbiosis, particularly involving *Porphyromonas gingivalis* (*P. gingivalis*) and *Fusobacterium nucleatum* (*F. nucleatum*), may contribute to the association between periodontitis and pancreatic cancer through specific mechanisms, with these microorganisms being investigated as candidate biomarkers for pancreatic cancer [13,14]. Additional evidence suggests that oral pathogens may interact with the intestinal microbiota and modulate local and systemic inflammatory responses, thereby potentially linking the proposed oral–gut–pancreatic axis to pancreatic cancer progression.

This review examines the evidence linking periodontitis to pancreatic cancer, with particular attention to microbial interactions along the proposed oral–gut–pancreatic axis. By integrating recent epidemiological, microbiome, and experimental evidence, it explores the possible mechanisms underlying this association and considers the potential clinical relevance of candidate microbial biomarkers and microbiota-targeted interventions.

In this narrative review, we searched PubMed and Web of Science for English-language articles available through September 2026. We focused primarily on studies published within the preceding 15 years, while retaining earlier landmark studies where relevant. The search combined terms related to periodontitis, periodontal disease, tooth loss, oral microbiota, gut microbiota, pancreatic microbiota, pancreatic cancer, and pancreatic ductal adenocarcinoma. Searches for specific microorganisms, including *Porphyromonas gingivalis* and *Fusobacterium nucleatum*, were combined with terms related to bacterial translocation, inflammation, immune regulation, microbial metabolites, biomarkers, prevention, and treatment. Human epidemiological and microbiome studies, together with experimental studies specific to pancreatic cancer, were prioritized. Studies of pancreatitis or other cancer models were considered when they provided relevant indirect mechanistic evidence. The literature was selected and organized around epidemiological associations, microbiota alterations, potential mechanisms, and clinical implications.

## Clinical research

Epidemiological studies investigating the association between periodontitis and pancreatic cancer have used a range of periodontal measures, including clinically diagnosed periodontitis, self-reported periodontal disease, tooth loss, and edentulism. Self-reported periodontal disease lacks clinical verification, whereas tooth loss and edentulism are nonspecific indirect measures because they may result from causes other than periodontitis. Despite these differences, observational studies suggest that these periodontal measures may be associated with pancreatic cancer risk. A large-scale retrospective cohort study using Taiwan's National Health Insurance Research Database reported that an insurance claims-based diagnosis of periodontal disease, including periodontitis, gingivitis, and other periodontal conditions, was associated with a 55% higher risk of pancreatic cancer (hazard ratio [HR] = 1.55, 95% confidence interval [CI] = 1.02–2.33) [15]. This association remained significant after adjusting for diabetes mellitus, allergies, hyperlipidemia, peptic ulcers, viral hepatitis, pancreatitis, chronic obstructive pulmonary disease, and alcohol-related disorders [15]. A recent meta-analysis of 8 observational studies (*N* = 476,245) reported a modest pooled association between periodontitis and pancreatic cancer risk (pooled HR = 1.56; 95% CI: 1.28-1.89) [16]. In a retrospective cohort study using a database of 1,000,000 enrollees from Taiwan's National Health Insurance program, Hwang IM et al. reported that periodontal treatment was associated with a 28% lower overall cancer risk (adjusted HR = 0.72, 95% CI = 0.68–0.76), including a 45% lower pancreatic cancer risk (adjusted HR = 0.55, 95% CI = 0.35–0.85) compared with no treatment [17].

Accumulating prospective evidence also supports an association between periodontitis and pancreatic cancer. In a register-based cohort study (*N* = 68,273), Heikkilä *P* et al. found that periodontitis was associated with overall cancer mortality (rate ratio [RR] = 1.33, 95% CI = 1.10–1.58), particularly pancreatic cancer-specific mortality (RR = 2.32, 95% CI = 1.31–3.98) [18]. A separate prospective cohort study of male health professionals aged 40–75 years (*N* = 51,529) found that participants who reported a history of periodontal disease had a higher risk of incident pancreatic cancer (adjusted RR = 1.64, 95% CI = 1.19–2.26) [12].

Beyond epidemiological associations, increasing attention has focused on the possible role of oral microbial dysbiosis in the relationship between periodontitis and pancreatic cancer. Early evidence from the European Prospective Investigation into Cancer and Nutrition cohort showed that individuals with elevated prediagnostic plasma antibody levels against *P. gingivalis* had an approximately twofold higher risk of developing pancreatic cancer than those with low antibody levels (OR = 2.14, 95% CI = 1.05–4.36) [13]. Subsequent oral microbiome sequencing studies further examined this association. Fan et al. applied bacterial 16S rRNA gene sequencing to prediagnostic oral wash samples from 361 pancreatic adenocarcinoma cases and 371 matched controls and found that carriage of *P. gingivalis* and *A. actinomycetemcomitans* was associated with 60% and 120% higher odds of pancreatic cancer, respectively (adjusted OR = 1.60, 95% CI: 1.15–2.22; OR = 2.20, 95% CI: 1.16–4.18) [19]. More recently, comprehensive profiling of the oral bacterial and fungal microbiome showed that broader patterns of oral microbial dysbiosis, rather than individual pathogens alone, were associated with subsequent pancreatic cancer risk [20]. Collectively, these findings suggest a possible link between periodontitis-associated oral microbial dysbiosis and pancreatic cancer development. Table 1 summarizes epidemiological studies investigating the association between periodontal conditions and pancreatic cancer.

**Table 1.** Epidemiological studies investigating the association between periodontal conditions and pancreatic cancer.

Literature	Study Type	Sample	Follow-up Duration	Periodontal Measure	Assessment Type	Main Findings
Heikkilä P, et al. [18]	Register-based prospective cohort study	68,273 adults	10 years	DMF, probing depth, CPI, tooth loss	Registry-based assessment	Higher pancreatic cancer-specific mortality among participants with periodontitis (RR = 2.32, 95% CI: 1.31–3.98)
Miskiewicz A, et al. [21]	Cross-sectional clinical study	120 adults	Apr 2012−Aug 2015	Plaque index, probing depth, CAL, BOP	Clinical examination	Higher periodontal inflammatory indices in pancreatic cancer patients (H = 24.94, *p* = 0.01)
Gerlovin H, et al. [22]	Prospective cohort study	38,986 African American women	2007−2016	Self-reported periodontal disease, tooth loss	Self-report	Higher pancreatic cancer risk with loss of ≥ 5 teeth (HR = 2.20, 95% CI: 1.11–4.33)
Hujoel PP, et al. [23]	Prospective cohort study	11,328 adults aged 25–74 years	Follow-up through 1992	Periodontal status and edentulism	Clinical examination	No statistically significant association between periodontitis and pancreatic cancer mortality (OR = 1.77, 95% CI: 0.85–3.67)
Stolzenberg-Solomon RZ, et al. [24]	Prospective cohort study	29,104 male smokers aged 50–69 years	1985−1997	Dentition history (0–10 missing teeth, 11–31 missing teeth, or edentulism)	Self-report	Higher pancreatic cancer risk with edentulism (HR = 1.63, 95% CI: 1.09–2.46)
Michaud DS, et al. [12]	Prospective cohort study	51,529 male healthcare professionals aged 40–75	1986−2002	History of periodontal disease with bone loss; number of natural teeth and tooth loss	Self-report	Higher pancreatic cancer risk associated with a history of periodontal disease (RR = 1.64, 95% CI: 1.19–2.26)
Huang J, et al. [25]	Prospective cohort study	19,924 adults	1973−2012	Number of teeth, dental plaque, and oral mucosal lesions	Clinical examination	Higher pancreatic cancer risk with higher dental plaque levels (HR = 2.10, 95% CI = 1.00–4.70)
Michaud DS, et al. [26]	Prospective cohort study	48,375 men	1986−2004	History of periodontal disease with bone loss and number of teeth	Self-report	Higher pancreatic cancer risk with a history of periodontal diseas (HR = 1.54, 95% CI = 1.16–2.04)
Arora M, et al. [27]	Prospective cohort study	15,333 Swedish twins	1963−2004	Self-report of tooth mobility, spontaneous tooth loss	Self-report	Higher pancreatic cancer risk with periodontal disease (HR = 2.06, 95% CI: 1.14–3.75)
Chang JS, et al. [15]	Retrospective cohort study	139,805 subjects with periodontal disease and 75,085 controls	1998−2009	Periodontal disease identified using ICD-9-CM codes	Registry-based assessment	Higher pancreatic cancer risk with periodontal disease (HR = 1.55, 95% CI: 1.02–2.33)

Abbreviations: DMF, decayed, missing, and filled teeth; CAL, clinical attachment loss; BOP, bleeding on probing; CPI, Community Periodontal Index; ICD-9-CM, International Classification of Diseases, Ninth Revision, Clinical Modification; HR, hazard ratio; RR, rate ratio or relative risk, as reported in the original studies; OR, odds ratio; CI, confidence interval. Note: Tooth mobility, tooth loss, and edentulism are nonspecific indirect measures of periodontal status.

## Microbiota in periodontitis and pancreatic cancer

Carcinogenic roles of microorganisms have been implicated in multiple malignancies [28]. Both periodontitis and pancreatic cancer have been associated with microbial dysbiosis. The following subsections summarize microbial compositional alterations and predominant taxa associated with periodontitis and pancreatic cancer (Figure 1).

**Figure 1.**
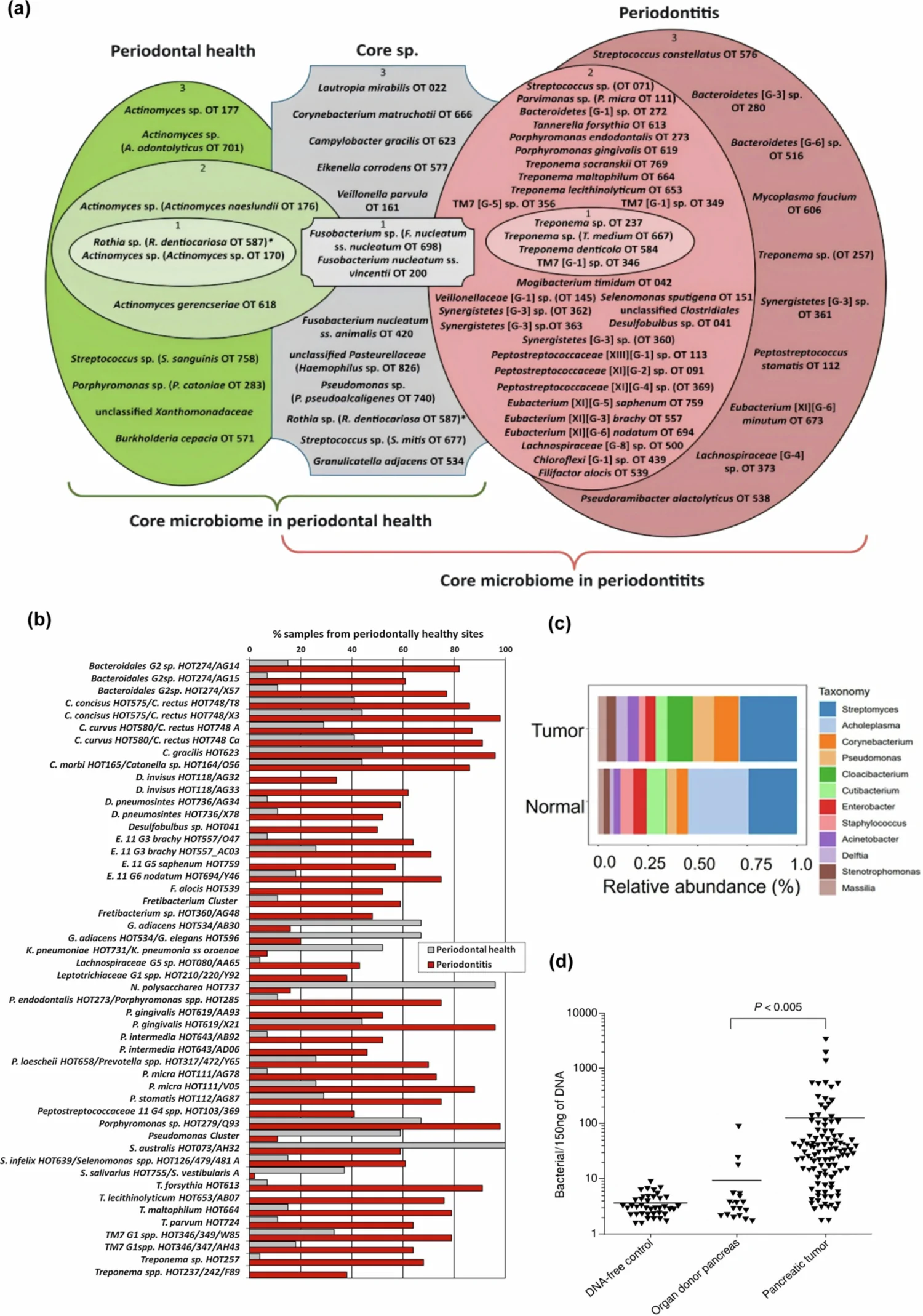
Microbial communities in healthy periodontium, periodontitis, and pancreatic tissues. (a) Composition of the core subgingival microbiome in health and periodontitis. Reproduced with permission [32]. Copyright 2013, The Authors. Published by *ISME J*. (b) Distinct microbial community profiles in healthy periodontium compared with periodontitis. Reproduced with permission [31]. Copyright 2014, The Authors. Published by *J Clin Periodontol*. (c) Relative abundances of predominant bacterial genera in human pancreatic tumors and matched adjacent histologically normal tissues [39]. Reproduced under the terms of the CC-BY Creative Commons Attribution 4.0 International license (https://creativecommons.org/licenses/by/4.0). (d) Bacterial presence was assessed by 16S ribosomal DNA quantitative polymerase chain reaction in three sample groups: ‘DNA-free control,’ ‘organ donor pancreas,’ and ‘pancreatic tumor.’ Reproduced with permission [40]. Copyright 2017, The Authors. Published by Science.

### Microbiota and periodontitis

Oral microbial homeostasis serves as an important ecological barrier for maintaining periodontal health, whereas disruption of its structure and function is closely associated with the onset and progression of periodontitis [29,30]. Compared with healthy periodontal conditions, periodontitis is characterized by increased microbial biomass and altered microbial diversity, accompanied by shifts in microbial community structure with emergence of newly dominant taxa without complete replacement of health-associated species [31,32]. *Treponema denticola* (*T. denticola*), *Tannerella forsythia* (*T. forsythia*), and *P. gingivalis* are significantly more abundant in patients with periodontitis [32,33]. The periodontitis-associated microbiome also contains elevated proportions of *Spirochaetes*, *Synergistetes*, *Firmicutes*, and *Chloroflexi* [32]. Recent studies have further identified additional periodontitis-associated species: *Bulleidia extructa* (*B. extructa*), *Lachnospiraceae bacterium oral taxon 096*, *Enterococcus casseliflavus* (*E. casseliflavus*), and *Campylobacter rectus* (*C. rectus*) [34]. The ecological characteristics of periodontitis-associated dysbiosis are further supported by the concept of periodontal microbial complexes. Among these, the classical ‘red complex’, consisting of *P. gingivalis*, *T. forsythia*, and *T. denticola*, is strongly associated with periodontal tissue destruction [35]. While the pathogenic mechanisms of *T. forsythia* remain less extensively investigated than those of other oral pathogens, several virulence-associated components, including the S-layer surface glycoprotein, may modulate host immune responses and influence T helper 17 (Th17)-related inflammatory pathways, thereby enhancing bacterial persistence [36]. *T. forsythia* pathogenicity is augmented through synergistic interactions with *P. gingivalis* and *T. denticola*, which may further contribute to alveolar bone loss [37]. Moreover, several studies have identified additional periodontitis-associated bacteria, including *Filifactor alocis* and *Dialister pneumosintes* [38]. Increasing periodontitis severity, reflected by deeper periodontal pockets and related periodontal indices, is associated with aggravated microbial dysbiosis, which has also been linked to systemic inflammatory disorders such as inflammatory bowel disease, hypertension, and diabetes [28].

### Microbiota and pancreatic cancer

Given the accumulating evidence linking the microbiota to pancreatic cancer susceptibility, tumor initiation, progression, and therapeutic response, the study of microbial communities associated with pancreatic cancer has emerged as an important area of research with potential implications for microbiota-based precision oncology strategies, although the underlying mechanisms remain under active investigation (Figure 2) [41].

**Figure 2.**
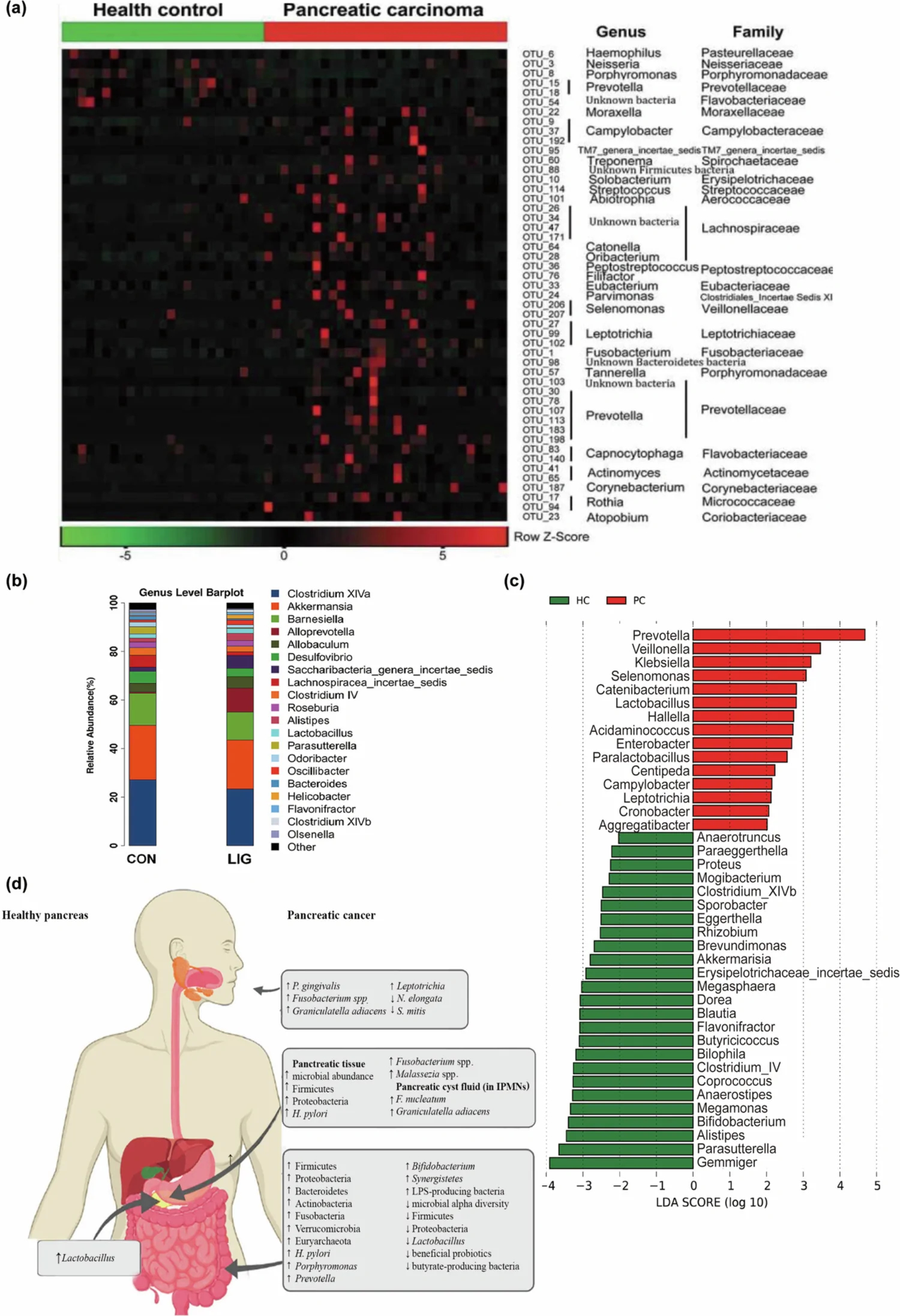
Representative oral, gut, and pancreatic microbiota profiles relevant to periodontitis and pancreatic cancer. (a) Heatmap showing tongue-coating microbial profiles in patients with pancreatic cancer and healthy controls. Reproduced under the Creative Commons Attribution 4.0 International license (https://creativecommons.org/licenses/by/4.0) [45]. Copyright 2019, The Authors. Published by *J Oral Microbiol*. (b) Genus-level composition of the gut microbiota in control (CON) and ligature-induced periodontitis (LIG) ApoE^-^/^-^ mice. Reproduced with permission [46]. Copyright 2022, The Authors. Published by *J Clin Periodontol*. (c) Differentially abundant fecal microbial genera in patients with pancreatic cancer (PC) and healthy controls (HC). Reproduced under the Creative Commons Attribution 3.0 International license (https://creativecommons.org/licenses/by/3.0/) [47]. Copyright 2017, The Authors. Published by *Oncotarget*. (d) Schematic summary of bacterial taxa reported to differ in the oral cavity, pancreatic tissue, and gut in pancreatic cancer, based on findings from multiple studies. Reproduced under the Creative Commons Attribution 4.0 International license (https://creativecommons.org/licenses/by/4.0) [6]. Copyright 2021, The Authors. Published by *Int J Mol Sci*.

The pancreas has historically been considered a sterile organ [40]. Whether the healthy pancreas harbors a stable resident microbiota remains unclear because pancreatic tissues are low-microbial-biomass environments susceptible to contamination during sampling and sequencing. Therefore, current studies primarily support the presence of microbial communities in diseased pancreatic tissues rather than definitively establishing a core microbiota in the healthy pancreas. Early studies reported substantially higher bacterial loads in PDAC tissues than in healthy donor pancreata [42]. In a landmark *Science* study, Geller LT et al. detected bacterial 16S rDNA by quantitative polymerase chain reaction in 86 of 113 (76%) PDAC specimens compared with 3 of 20 (15%) normal pancreatic controls (*P* < 0.005) [40]. Metagenomic profiling further revealed differences in the relative abundances of predominant bacterial genera between human pancreatic tumors and matched adjacent histologically normal tissues [39]. Pushalkar S et al. identified distinct microbial signatures, with higher abundances of *Brevibacterium*, *Brevibacteriaceae*, *Sphingobacteriaceae*, *Chlamydiales*, *Chlamydiae*, and *Methylobacteriaceae* detected in normal pancreatic tissues [43]. In contrast, PDAC samples demonstrated a 1,000-fold increase in pancreatic bacterial loads, with significant enrichment of *Sphingobacteriales*, *Sphingobacteriia*, *Chlamydia*, *Mogibacterium*, and *Oscillospira*[41,43]. In the same study, 16S rRNA gene sequencing of PDAC tumor samples from 12 patients identified 13 distinct bacterial phyla, with Proteobacteria (45%), Bacteroidetes (31%), and Firmicutes (22%) being the most abundant [43]. Preclinical studies suggest that pancreatic bacterial expansion in PDAC may promote carcinogenesis through pro-inflammatory responses, immune microenvironment remodeling, direct mutagenesis, or activation of oncogenic signaling pathways [35]. Thomas RM et al. provided complementary culture-based evidence: aerobic and anaerobic culturing of lysates from independent malignant pancreatic specimens yielded viable *Corynebacterium*, *Enterobacter*, and *Escherichia* species, supporting the presence of cultivable bacteria in the pancreatic tissues [44]. Collectively, current evidence supports the presence of microbial signals in pancreatic tissues; however, whether the pancreas harbors a stable resident microbiota remains incompletely understood [44].

### Oral–gut microbiota and pancreatic cancer

The oral cavity and gastrointestinal tract constitute an ecological continuum due to anatomical continuity, with ectopic colonization of their microbiota representing an important component of the proposed ‘oral–gut microbiome axis.’ Beyond localized oral pathologies such as periodontitis, oral dysbiosis has also been investigated in relation to extraoral diseases, including pancreatic cancer, with systemic inflammation and immune dysregulation proposed as potential underlying mechanisms (Figure 3) [28].

**Figure 3.**
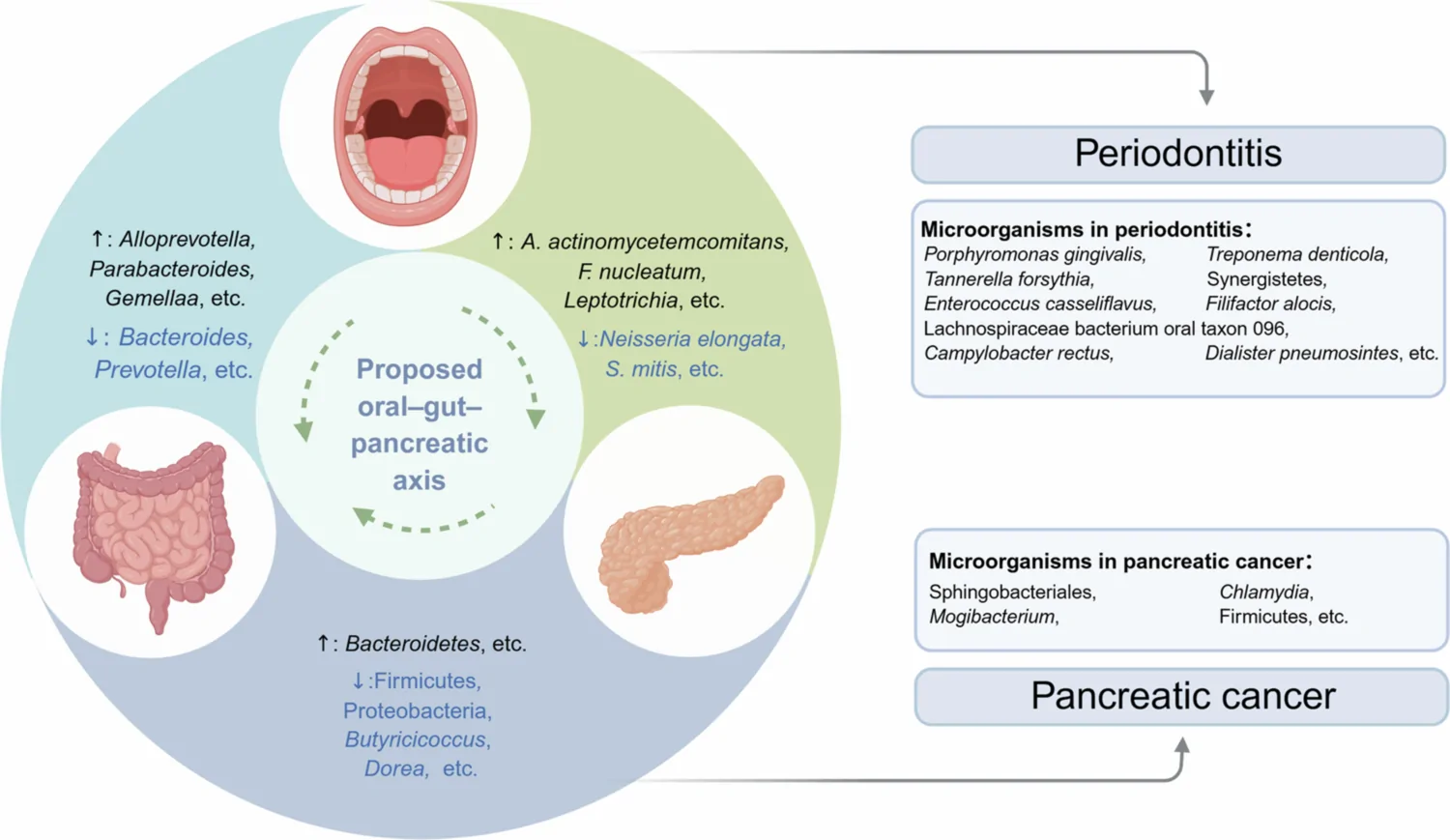
Overview of reported oral, gut, and pancreatic microbiota alterations associated with periodontitis and pancreatic cancer. Findings from separate studies indicate that periodontitis-related gut microbiota alterations include enrichment of *Alloprevotella* and lower relative abundance of *Bacteroides*. Pancreatic cancer-associated oral microbial features include *A. actinomycetemcomitans*, *F. nucleatum*, *Leptotrichia*, *N. elongata*, and *S. mitis*, whereas gut microbiota alterations include increased relative abundance of Bacteroidetes and decreased relative abundance of Firmicutes. Representative microbial taxa associated with periodontitis and detected in pancreatic cancer tissues are also shown. Dashed arrows indicate proposed interactions among the oral, gut, and pancreatic microbiota. Created in BioRender. J, S. (2026) https://BioRender.com/k92fyyj.

Studies using different oral sample types have reported pancreatic cancer-associated differences in several bacterial taxa. These findings include the detection or increased abundance of *Aggregatibacter actinomycetemcomitans* (*A. actinomycetemcomitans*), *F. nucleatum*, and *Leptotrichia*, as well as a lower abundance of *Neisseria elongata* (*N. elongata*), *Streptococcus mitis* (*S. mitis*), and *Bacteroides* species [6,19,45,48]. Notably, specific microbial signatures exhibit diagnostic potential: the combination of *N. elongata* and *S. mitis* discriminates patients with pancreatic cancer from HC, while elevated *Leptotrichia*-to-*Porphyromonas* ratios represent potential early-detection biomarkers [6,48]. Accumulating evidence suggests that oral dysbiosis may be associated with pancreatic cancer pathobiology [19,49,50]. In mouse models, oral administration of *P. gingivalis* was associated with pancreatic acinar-to-ductal metaplasia and pancreatic microbiome reprogramming, accelerating progression from pancreatic intraepithelial neoplasia (PanIN) to PDAC [50]. In addition to differences in bacterial composition, bacterial 16S DNA quantity and detection frequency in pancreatic cyst fluid were associated with lesion severity. Gaiser RA et al. reported a stepwise increase in the proportion of bacterial DNA-positive cyst fluid samples, from 33.0% in non-IPMN lesions to 59.6% in intraductal papillary mucinous neoplasm (IPMN) lesions and 81.5% in the cancer group [51].

The oral cavity, serving as a dynamic microbial reservoir, may influence gut microbiota homeostasis through swallowing and hematogenous dissemination [28]. Accumulating evidence suggests that periodontitis is associated with compositional shifts in gut microbial profiles. Li L et al. compared the intestinal microbiota between ligature-induced periodontitis (LIG) and control (CON) ApoE−/− mice and found significant gut microbiome alterations, characterized by enrichment of *Alloprevotella*, *Parabacteroides*, *Turicibacter*, and *Gemella* in the LIG group [46]. Notably, Sato K et al. reported that oral administration of *P. gingivalis* to mice reduced the relative abundance of *Bacteroidetes* while increasing the proportion of *Firmicutes* in gut microbiota [52]. Furthermore, Kramer et al. demonstrated in ApoE−/− mice that *P. gingivalis* colonization decreased gut bacterial community complexity, as measured by alpha-diversity indices, suggesting that *P. gingivalis* may modulate gut microbiome composition [53]. A large-scale study using separately collected salivary and fecal samples from individuals with and without periodontal disease demonstrated associations between periodontal status and oral–gut microbial configurations [54]. Additionally, a longitudinal analysis by Baima G et al. of paired saliva and fecal samples collected before and after periodontal therapy showed that treatment was accompanied by improvements in oral dysbiosis and changes in gut microbial composition [55].

Emerging evidence indicates associations between gut microbiota alterations and pancreatic cancer. Ren Z et al. reported a significantly higher abundance of *Bacteroidetes* (*P* < 0.001) and decreased abundance of *Firmicutes* and *Proteobacteria* (both *P* < 0.05) in fecal samples from patients with pancreatic cancer compared with healthy controls [47]. Together with the studies of periodontitis-related gut microbial alterations described above, these findings provide indirect support for a possible connection along the proposed oral–gut–pancreatic axis. Nevertheless, direct evidence linking periodontitis-associated gut microbial alterations to pancreatic carcinogenesis remains limited, and the underlying mechanisms require further investigation.

## Potential pathways linking bacteria to pancreatic cancer

As discussed above, accumulating evidence suggests that oral microbiota may contribute to the initiation and progression of pancreatic cancer. Key proposed mechanisms underlying this possible association include bacterial translocation and the effects of bacterial virulence factors (Figure 4a,b).

**Figure 4.**
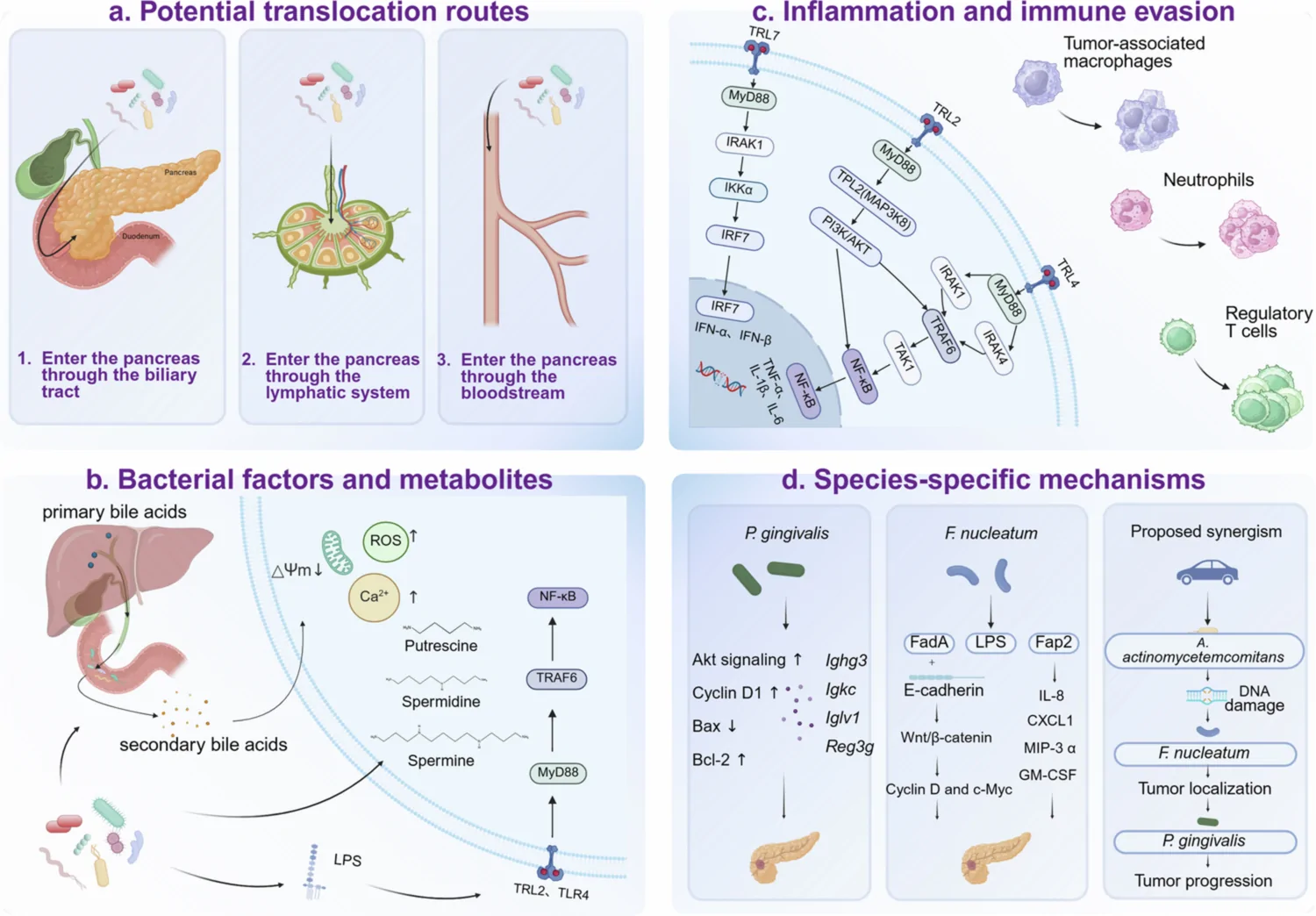
Potential routes and mechanisms by which bacteria may influence pancreatic cancer. (a) Potential routes of bacterial translocation to the pancreas include retrograde entry through the biliary tract and dissemination via the lymphatic system or bloodstream. (b) Bacterial factors and microbiota-associated metabolites, including lipopolysaccharide (LPS), polyamines, and secondary bile acids, may modulate signaling pathways relevant to pancreatic cancer. (c) Bacteria may influence inflammatory responses and immune evasion through TLR-related signaling and modulation of tumor-associated macrophages, neutrophils, and regulatory T cells. (d) Representative species-specific mechanisms involving *P. gingivalis* and *F. nucleatum*, together with a proposed synergistic model, are shown. Created in BioRender. J, S. (2026) https://BioRender.com/zo75wsw.

### Bacterial translocation

Bacterial translocation refers to the process by which bacteria and their metabolic products traverse anatomical barriers, including mucosal surfaces and vascular endothelium, and disseminate from their native ecological niches, such as the gut and oral cavity, to extraoral sites or the systemic circulation. This process is often associated with disruption of local barrier integrity and may contribute to systemic infections or inflammatory responses. Oral diseases, such as periodontitis, have been associated with multiple systemic conditions, including cardiovascular disease and pancreatic cancer [56]. Recently, the potential role of bacterial translocation in pancreatic cancer has received substantial attention in tumor immunology and microbiology research. Michaud DS et al. proposed several potential routes by which oral bacteria may reach the pancreas, including the systemic circulation, bile duct, duodenum, and lymphatic system [57]. Experimental murine studies suggest that periodontal pathogens, particularly *P. gingivalis*, may disseminate beyond the oral cavity and may contribute to pancreatic microbial dysbiosis and tumor progression through multiple possible routes, including hematogenous and gastrointestinal dissemination [43,50,58]. Collectively, these findings support a proposed dual-translocation hypothesis, in which oral bacteria or their products may reach the pancreas via the oral–gut axis through the biliary tract (Type I) or via the circulatory system through hematogenous dissemination (Type II). Current evidence raises the possibility that bacterial translocation through these routes may be involved in pancreatic tumorigenesis, progression, and immune evasion (Figure 4a).

#### Oral–gut axis

Due to the anatomical continuity of the oral–gut axis, oral bacteria may influence the composition of the gut microbiota through direct and indirect mechanisms [28]. Anatomically, the pancreatic duct drains pancreatic secretions into the duodenum through the sphincter of Oddi, which regulates the flow of pancreatic juice and bile. Under physiological conditions, the sphincter remains contracted in the interdigestive state, thereby limiting reflux of intestinal content into the pancreatic duct. Following food intake, sphincter relaxation permits the controlled passage of pancreatic juice into the duodenum [59]. Sphincter of Oddi dysfunction (SOD) may predispose individuals to acute pancreatitis through aberrant pancreatobiliary flow dynamics, and evidence indicates an association between SOD and pancreatitis [60]. In the setting of impaired sphincter function or abnormal pancreatobiliary flow, retrograde entry of intestinal bacteria into the pancreas has been proposed as a potential route of microbial translocation. Such translocation may alter the pancreatic microbial environment and expose pancreatic tissues to microbial toxins and metabolites, potentially affecting processes relevant to pancreatic cancer development and progression. Moreover, the immunosuppressive milieu characteristic of pancreatic cancer may further favor the persistence of bacteria or bacterial products within the pancreatic tumor microenvironment, which could influence tumor progression and clinical outcomes [61]. A 2020 report published in *Science* identified *Proteobacteria* as a dominant phylum within the pancreatic cancer-associated microbiome, a finding consistent with the possibility of retrograde migration from the duodenum to the pancreatic duct [62].

Recent experimental studies further support the possibility that bacteria can enter the pancreas through oral–gut-related routes and influence pancreatic tumor progression. Pushalkar et al. demonstrated that orally administered fluorescently labeled gut bacteria were detected in the pancreas of mice, supporting gut-to-pancreas microbial translocation [43]. More directly, Saba et al. showed that the oral periodontal pathogen *P. gingivalis* migrated from the oral cavity to the pancreas in mice and accelerated PanIN-to-PDAC progression [50]. In addition, Tan et al. detected *P. gingivalis* in both the oral samples and pancreatic tumor tissues from patients with pancreatic cancer. In mouse models, oral administration of *P. gingivalis* accelerated pancreatic tumor growth through neutrophil elastase derived from tumor-associated neutrophils [58].

#### Circulatory system

Bacteria originating from the oral cavity may influence pancreatic pathophysiology not only through gastrointestinal routes but also via hematogenous dissemination. In the context of periodontitis or other oral inflammatory conditions, bacterial infections elicit immune responses that induce the release of pro-inflammatory cytokines, including interleukin (IL)-1 and tumor necrosis factor-*α* (TNF-*α*), as well as matrix metalloproteinases (MMPs), such as MMP-2 and MMP-9. These mediators compromise tight junction integrity between epithelial cells, increase barrier permeability, and impair mucosal repair through epithelial apoptosis and dysfunction [63]. Simultaneously, localized inflammation enhances oral mucosal vascular permeability, facilitating hematogenous dissemination of oral pathogens, such as *A. actinomycetemcomitans* and *P. gingivalis*. Oral bacteria can access the systemic circulation through multiple routes, including disruption of barrier integrity along the oral–gut axis, providing a possible route by which oral pathogens may reach distant organs, including the pancreas. Li X et al. reviewed evidence of bacteremia following dental procedures, such as periodontal surgery and root planing, indicating that oral microbiota can transiently enter the bloodstream after dental manipulation [64]. A meta-analysis by Carolina C. Martins et al., encompassing 64 non-randomized and 25 randomized controlled trials, reported the highest bacteremia incidence after tooth extraction (62%–66%), followed by scaling/root planing (44%–36%) and routine oral hygiene procedures (27%–28%) [65]. Furthermore, oral bacteria may interact with vascular endothelium to gain systemic access. Hayashi C et al. reviewed evidence suggesting that *P*. gingivalis can disseminate through the circulation and interact with platelets and vascular endothelial cells. Mechanistically, its major and minor fimbriae may mediate binding to lipid raft-associated receptors, including CAMs, TLRs, and CD36, thereby inducing endothelial responses characterized by cytokine, chemokine, and surface molecule production [66]. Complementing these findings, Xi M et al. summarized that periodontal pathogens may disseminate hematogenously following disruption of barrier integrity along the oral–gut axis [28].

The lymphatic system represents an additional possible pathway for bacterial translocation. Bacteria and bacterial products, including lipopolysaccharide (LPS) and peptidoglycans, may cross a disrupted intestinal barrier and reach the mesenteric lymph nodes. This process may promote systemic dissemination of intestinal bacteria and their cell wall components, potentially leading to sepsis and multiple organ failure. Fritz S et al. demonstrated that selective digestive decontamination of the small intestine markedly reduced infections in mesenteric lymph nodes, subsequently decreasing secondary pancreatic infections [67]. They concluded that bacterial translocation occurred via mesenteric lymph nodes rather than through hematogenous or peritoneal routes [67]. Accordingly, this animal study suggests that intestinal bacteria may disseminate through mesenteric lymphatic pathways to the pancreas [67].

### Effects of bacterial virulence factors and microbial metabolites

Bacteria may influence host responses through virulence factors, structural components, and microbial metabolites. Bacterial components, including LPS, may engage pattern recognition receptors (PRRs), including TLRs, whereas microbial metabolites such as short-chain fatty acids (SCFAs) and polyamines may modulate host immune responses through distinct signaling pathways (Figure 4b). These factors may influence biological processes relevant to pancreatic cancer development and progression.

#### LPS

In the gut microbiota of patients with pancreatic cancer, the proportion of LPS-producing bacteria is elevated [47]. LPS, a bacterial component implicated in pancreatic cancer, constitutes part of the outer membrane of Gram-negative bacteria and may be released into the surrounding microenvironment through outer membrane vesicles or bacterial cell wall degradation. Hayashi M et al. conducted immunohistochemical analyses using antibodies against bacterial LPS on pancreatic cancer and normal pancreatic tissue sections, detecting LPS in tumor tissues but not in normal pancreatic tissues [68]. LPS has been implicated in pancreatic cancer progression by mediating and sustaining TLR-induced inflammation [6]. Specifically, *P. gingivalis*-derived LPS can participate in host innate immune recognition through TLR2 and TLR4 [69]. In pancreatic cancer cells, LPS has been shown to enhance cellular invasiveness through TLR4/MyD88-dependent signaling; inhibition of the NF-κB pathway attenuates the LPS-dependent increase in invasion, and siRNA-mediated suppression of TLR4 or MyD88 similarly reduces LPS-induced invasiveness [70]. Collectively, these findings indicate that LPS-induced TLR signaling may contribute to inflammatory signaling and invasive behavior in pancreatic cancer.

Beyond its immunomodulatory effects, LPS may directly affect epithelial cells, including neoplastic cells, and promote tumor-associated phenotypic changes. NF-κB is a transcription factor regulating pro-inflammatory, pro-survival, and pro-metastatic gene programs [71,72]. Experimental studies have shown that LPS can activate NF-κB-related signaling in pancreatic cancer cells, while NF-κB activation has been implicated in pancreatic cancer cell proliferation and tumor progression [73,74].

#### Bacterial metabolites

Bacterial metabolites are increasingly recognized as modulators of pancreatic cancer progression. These microbe-derived molecules may exert systemic effects through circulation, with principal mediators including SCFAs and polyamines. Oral and intestinal bacteria produce SCFAs, such as acetate, propionate, and butyrate, through dietary fiber fermentation; these metabolites have been associated with anti-inflammatory and anti-neoplastic properties [75]. Within pancreatic cancer-associated microbial communities, reduced abundance of probiotic bacteria and butyrate-producing species may indicate impaired SCFA-mediated regulatory activity [76]. Huang Y et al. reported a significantly higher *Megasphaera* genus abundance (*P* ≤ 0.001) in the intratumoral microbiome of PDAC long-term survivors with postoperative survival exceeding 600 days compared with short-term survivors (STS) with survival between 120 and 300 days [77]. *Megasphaera sp.* XA511, a strain isolated from the feces of a healthy donor, produced butyrate, valerate, and isovalerate, whereas the bacterial species examined from STS-enriched genera primarily produced formate and acetate. These findings suggest that *Megasphaera* may enhance responses to programmed cell death protein 1 (PD-1) blockade through immunomodulatory metabolites, as further supported by in vivo validation in a 4T1 tumor-bearing mouse model [77].

Other microbiota-associated metabolites implicated in pancreatic cancer include polyamines, which are polycationic alkylamines such as putrescine, spermidine, and spermine. These molecules participate in multiple oncogenic and cellular signaling pathways, and their depletion induces cell cycle arrest. In a genetically engineered mouse model of PDAC, serum polyamine concentrations increased significantly with tumor progression [78]. Elevated serum polyamine concentrations were also observed in patients with PDAC compared with healthy controls [78]. Polyamines may represent therapeutic targets or potential biomarkers for early pancreatic cancer detection [6].

Secondary bile acids, another class of bacterial metabolites, are implicated in pancreatic cancer development. These metabolites are generated through gut microbiota-mediated modification of primary bile acids. Primary bile acids, such as cholic acid, are synthesized in the liver and conjugated with glycine or taurine before biliary secretion and transport to the duodenum. Gut bacteria, including certain *Clostridium* species, mediate bile acid dehydroxylation and deconjugation, thereby converting primary bile acids into secondary bile acids, such as deoxycholic acid and ursodeoxycholic acid (UDCA) [76]. A study involving 15 patients with pancreatic cancer and 15 with benign disease reported higher overall levels of detected bile acid species in patients with pancreatic cancer [79]. Bile acids are also associated with established pancreatic cancer risk factors, including pancreatitis and gallstones [80,81]. Muili et al. proposed that bile acids induce acinar cell injury and pancreatitis by reducing mitochondrial membrane potential and increasing reactive oxygen species (ROS) production, thereby triggering cytosolic Ca^2+^ signaling in acinar cells [80,82,83]. Furthermore, Tucker et al. reported that bile acids upregulate cyclooxygenase-2 messenger RNA expression in pancreatic cancer cells, suggesting a possible role for bile acids in pancreatic cancer pathogenesis [84]. As reviewed by Kiss et al., bile acids may activate oncogenic signaling pathways in pancreatic cancer cells by interacting with the bile acid receptor Takeda G-protein-coupled receptor 5, resulting in activation of the epidermal growth factor receptor, MAPK, and signal transducer and activator of transcription 3 (STAT3) signaling pathways, which promote cell cycle progression [76]. Notably, certain bile acids, including UDCA, may exert anti-neoplastic effects by inhibiting epithelial–mesenchymal transition (EMT) in pancreatic cancer cell lines [76].

## Mechanisms of bacterial regulation in pancreatic cancer

Bacteria associated with the oral–gut axis may influence pancreatic cancer through interconnected inflammatory and immunomodulatory mechanisms, while specific bacterial species may further exert distinct effects on tumor-related processes through species-dependent molecular interactions (Figure 4c,d). These mechanisms provide a possible biological basis for understanding how bacterial dysbiosis may contribute to pancreatic cancer initiation and progression. Representative mechanistic studies, including the experimental models used and their relevance to pancreatic cancer, are summarized in Table 2.

**Table 2.** Representative mechanistic studies relevant to the proposed association between periodontitis and pancreatic cancer.

Evidence relevance	Literature	Study design/model	Proposed mechanism	Main Findings
Pancreatic cancer-specific evidence	Pushalkar et al. [39].	Human PDAC tissues and mouse PDAC models	Microbial translocation and immune regulation	Human PDAC tissues had higher bacterial abundance; in mice, microbial ablation reduced immunosuppression and tumor progression.
Pancreatic cancer-specific evidence	Saba et al. [49].	iKC mice and human PanIN samples	Oral-to-pancreas translocation	*P. gingivalis* was detected in human PanIN; in mice, it reached the pancreas and accelerated PanIN-to-PDAC progression.
Pancreatic cancer-specific evidence	Tan et al. [57].	Human oral and pancreatic samples and mouse models	Neutrophil-mediated tumor promotion	*P. gingivalis* was detected in human samples and promoted pancreatic tumor growth through neutrophil elastase in mice.
Pancreatic cancer-specific evidence	Ikebe et al. [69].	Pancreatic cancer cell lines	LPS–TLR4/MyD88/NF-κB signaling	LPS enhanced pancreatic cancer cell invasion through TLR4/MyD88/NF-κB signaling.
Pancreatic cancer-specific evidence	Mendez et al. [77].	PDAC mice and human serum samples	Microbial polyamine metabolism	Polyamine biosynthesis increased during PDAC progression in mice, with elevated serum polyamines also found in patients.
Pancreatic cancer-specific evidence	Gnanasekaran et al. [85].	Pancreatic cancer cells and a xenograft model	Intracellular bacterial persistence	Intracellular persistence of *P. gingivalis* enhanced Akt signaling, cell proliferation, and xenograft growth.
Pancreatic cancer-specific evidence	Udayasuryan et al. [86].	Pancreatic cancer cell lines	Fap2-dependent infection and cytokine signaling	*F. nucleatum* promoted cytokine release, proliferation, and migration in pancreatic cancer cells.
Pancreatic cancer-specific evidence	Hayashi et al. [67].	Human pancreatic tissues, cells, and mouse models	CXCL1–CXCR2 signaling	*F. nucleatum* DNA was detected in human tumors; experimental models implicated CXCL1–CXCR2 signaling in tumor promotion.
Supporting evidence from other models	Li et al. [46].	Ligature-induced periodontitis in ApoE^-^/^-^ mice	Periodontitis-associated gut dysbiosis	Experimental periodontitis altered gut microbiota and systemic inflammatory responses in mice.
Supporting evidence from other models	Sato et al. [51]; Kramer et al. [52].	Arthritis and atherosclerosis mouse models	Oral pathogen-induced gut dysbiosis	Oral exposure to *P*. gingivalis altered gut microbiota and inflammatory responses in mice.
Supporting evidence from other models	Fritz et al. [66].	Acute necrotizing pancreatitis rat model	Intestinal bacterial translocation	Small-intestinal decontamination reduced bacterial translocation and pancreatic infection in rats.
Supporting evidence from other models	Binder Gallimidi et al. [87].	Oral cancer cells and mouse model	TLR2–IL-6–STAT3 signaling	*P. gingivalis* and *F. nucleatum* promoted inflammatory signaling and oral tumor progression.
Supporting evidence from other models	Hiraki et al. [88]; Yin et al. [89].	LPS exposure and chronic pancreatitis models in mice	Reg3-associated pancreatic effects	*P. gingivalis* LPS increased pancreatic Reg3 expression, while Reg3g promoted carcinogenesis in a separate mouse model.
Supporting evidence from other models	Parhi et al. [90]; Abed et al. [91].	Breast and colorectal cancer models	Fap2-mediated tumor localization	Fap2 mediated *F. nucleatum* localization to Gal-GalNAc-expressing tumors.

Abbreviations: CXCL1, C-X-C motif chemokine ligand 1; CXCR2, C-X-C chemokine receptor type 2; LPS, lipopolysaccharide; MyD88, myeloid differentiation primary response protein 88; NF-κB, nuclear factor kappa-B; PanIN, pancreatic intraepithelial neoplasia; PDAC, pancreatic ductal adenocarcinoma; TLR, Toll-like receptor.

Oral–gut axis bacteria may promote pancreatic cancer through inflammatory and immunomodulatory mechanisms, including TLR activation, altered immune-cell infiltration, and immune evasion (Figure 4c). Bacteria-induced inflammation may reshape tumor-associated immune cells, including regulatory T cells (Tregs), macrophages, and neutrophils, thereby promoting an immunosuppressive tumor microenvironment. Wang X et al. reported systemic Th17/Treg imbalance in patients with pancreatic cancer, characterized by increased Treg proportions, reduced Th17 cell proportions, and altered cytokine levels, including IL-10, IL-23, IL-17, and interferon-gamma (IFN-*γ*) [92]. Pushalkar et al. further showed that gut microbiota ablation enhanced pancreatic cancer immunogenicity in murine models by promoting CD4^+^ T helper 1 polarization, increasing CD8^+^ T-cell infiltration, and reducing myeloid-derived suppressor cell accumulation [43]. As summarized in recent reviews, microbiome-associated regulation of tumor-associated macrophage phenotypes may suppress CD4+ and CD8+ T-cell activation within the PDAC tumor microenvironment [93]. In addition, Tan Q et al. reported that C-X-C chemokine receptor type 2 (CXCR2) blockade attenuated tumor-promoting effects in *P. gingivalis*-exposed subcutaneous pancreatic cancer models, suggesting that *P. gingivalis* may recruit tumor-associated neutrophils and promote a pro-inflammatory and immunosuppressive tumor microenvironment [58].

During pancreatic cancer pathogenesis, oral-gut axis bacteria may activate TLR signaling, particularly TLR2 and TLR4, by engaging pathogen-associated molecular patterns (PAMPs) such as bacterial LPS. Binding of PAMPs to TLRs activates myeloid differentiation primary response protein 88 (MyD88)/nuclear factor kappa-B (NF-κB) signaling, thereby inducing pro-inflammatory cytokine release and immune activation [94]. Although these responses contribute to pathogen clearance, TLR signaling can also be associated with anti‑apoptotic effects and tumor‑promoting inflammation in chronic contexts [95]. Previous studies have shown that *P. gingivalis*-derived LPS can interact with TLR2 and TLR4 [66,69,96]. Bacterial LPS may also enhance the invasive ability of pancreatic cancer cells through TLR4/MyD88 signaling [96]. TLR7 is minimally expressed in normal pancreatic tissues but aberrantly expressed in pancreatic cancer, where TLR7 activation may promote tumor growth and early carcinogenic gene-expression changes, including PTEN loss and PI3K/AKT pathway activation [96,97]. TLR-mediated NF-κB activation stimulates secretion of pro-inflammatory cytokines, including TNF-*α*, IL-1β, and IL-6, thereby fostering local inflammation and systemic inflammatory cascades that may contribute to pancreatic cancer progression. Collectively, microbial components may influence pancreatic cancer-related inflammatory and immune responses through TLR-associated signaling pathways, including MyD88, PI3K/AKT, NF-κB, and mitogen-activated protein kinase (MAPK) signaling [49,85,95–97].

Emerging evidence suggests that specific oral bacteria may exert distinct tumor-promoting effects in pancreatic cancer (Figure 4d). The following section discusses representative bacterial species, including *P. gingivalis* and *F. nucleatum*, and summarizes their potential roles and underlying mechanisms in pancreatic cancer development (Figure 5).

**Figure 5.**
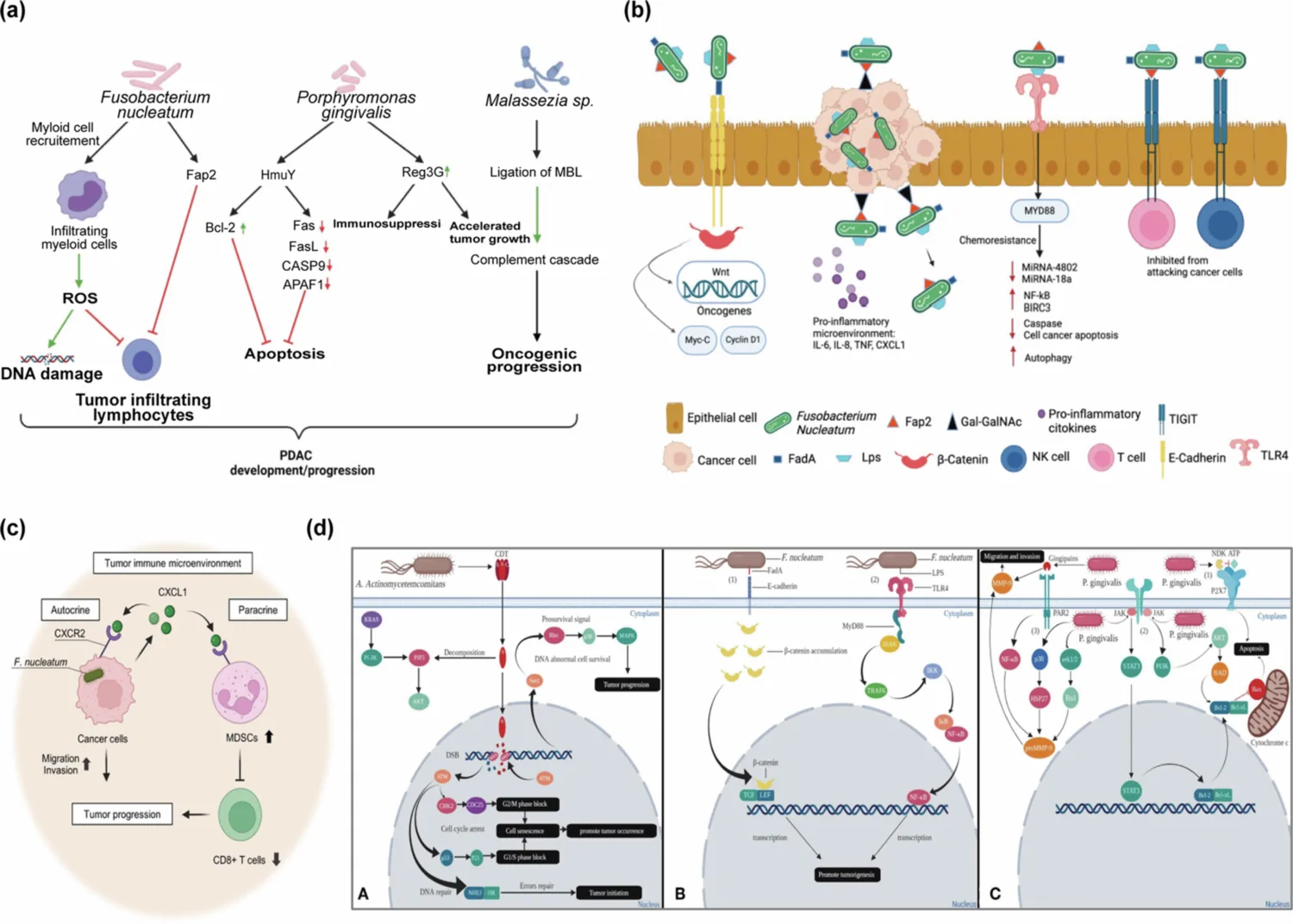
Potential mechanisms by which distinct microorganisms influence pancreatic cancer. (a) Overview of potential mechanisms involving *F. nucleatum*, *P. gingivalis*, and *Malassezia* species in PDAC development and progression. Licensed under the Creative Commons Attribution 4.0 International license (https://creativecommons.org/licenses/by/4.0) [103]. Copyright 2021, The Authors. Published by *Cancers (Basel)*. (b) Proposed effects of *F. nucleatum* virulence factors on tumor-related signaling and immune responses. Reproduced under the Creative Commons Attribution 4.0 International license (https://creativecommons.org/licenses/by/4.0) [90]. Copyright 2024, The Authors. Published by *Pathogens*. (c) Experimental findings showing that intratumoral *F. nucleatum* promotes pancreatic cancer progression through autocrine and paracrine CXCL1–CXCR2 signaling. Reproduced under the Creative Commons Attribution 4.0 International license (https://creativecommons.org/licenses/by/4.0) [68]. Copyright 2023, The Authors. Published by *Cancer Sci*. (d) Conceptual model of potential host-cell alterations induced by virulence factors from oral bacteria, including *P. gingivalis*, *F. nucleatum*, and *A. actinomycetemcomitans*. Reproduced under the Creative Commons Attribution 4.0 International license (https://creativecommons.org/licenses/by/4.0) [88]. Copyright 2019, The Authors. Published by *Front Cell Infect Microbiol*.

### P. gingivalis

*P. gingivalis*, a keystone periodontal pathogen, may promote pancreatic cancer progression through multiple molecular mechanisms. Saba E et al. reported significantly higher proportions of PanIN-3 and PDAC in the pancreatic tissues of iKC mice that were orally administered *P. gingivalis*, indicating accelerated malignant transformation from PanIN to PDAC in this Kras-driven iKC mouse model [50].

*P. gingivalis* may promote pancreatic cancer cell proliferation and tumor growth. Gnanasekaran J et al. co-cultured pancreatic cancer cells with *P. gingivalis* under hypoxic conditions, resulting in increased cellular proliferation (*P* ≤ 0.05). This proliferative effect was associated with the extent of bacterial intracellular persistence, enhanced Akt signaling, and increased cyclin D1 expression [87]. Notably, while *P. gingivalis* promotes oral squamous cell carcinoma growth via TLR2-dependent mechanisms [98], TLR2 neutralization did not attenuate *P. gingivalis*-induced PDAC cell proliferation, indicating that this proliferative effect was independent of TLR2 signaling [87].

Infection with *P. gingivalis* may modulate host cell gene expression, particularly genes regulating apoptosis, immune evasion, and the cell cycle. Previous studies have shown that *P. gingivalis* can modulate apoptosis-related proteins, including the anti-apoptotic protein Bcl-2 and the pro-apoptotic proteins Bax and Bcl-2-associated agonist of cell death (BAD) [88,99]. These effects involve Akt-mediated BAD phosphorylation, increased Bcl-2 expression, and reduced Bax expression, thereby suppressing mitochondria-dependent apoptosis [88,99]. A recent study examining pancreatic gene expression in mice that received intraperitoneal administration of *P. gingivalis*-derived LPS for 1 month found that several pancreatic cancer-associated genes were upregulated, including immunoglobulin heavy constant gamma 3, immunoglobulin kappa constant, immunoglobulin lambda variable 1, and regenerating islet-derived protein 3 gamma (*Reg3g*), despite the absence of overt inflammation [89]. Yin et al. further demonstrated that *Reg3g* may contribute to pancreatic cancer initiation and progression, potentially by enhancing the transcription of pancreatic tumor markers, suppressing anti-tumor immunity, and activating the STAT3/p65 signaling pathway [100]. Moreover, intracellular *P. gingivalis* has been proposed to interact with oncogenic alterations affecting related proliferative pathways, including mutant *KRAS*, a hallmark genetic alteration in PDAC [87].

### F. nucleatum

*F. nucleatum* can promote pancreatic cancer cell proliferation and migration through signal transduction pathways. It possesses several virulence factors, including Fusobacterium adhesion A (FadA), LPS, and Fusobacterium autotransporter protein 2 (Fap2). In colorectal cancer models, FadA binds E-cadherin, activating the Wnt/β-catenin pathway and upregulating cyclin D and c-Myc expression, thereby promoting tumorigenic progression [90]. Although this mechanism was characterized in colorectal cancer models, it provides a conceptual basis for investigating whether analogous adhesion-mediated pathways operate in PDAC. Adhesion of *F. nucleatum* to colorectal adenocarcinoma and breast cancer cells has been reported to depend on Fap2 [86,91]. In pancreatic cancer cells, a study reported that Fap2-deficient *F. nucleatum* showed significantly reduced infection of BxPC-3 and Panc-1 cells, suggesting that Fap2 may contribute to *F. nucleatum* infection of pancreatic cancer cells [101]. *F. nucleatum* infection increased the secretion of granulocyte-macrophage colony-stimulating factor (GM-CSF), C-X-C motif chemokine ligand 1 (CXCL1), IL-8, and macrophage inflammatory protein 3-alpha in pancreatic cancer cells [101]. GM-CSF may be associated with pancreatic cancer cell proliferation, whereas CXCL1 has been associated with metastasis and chemoresistance [90]. These cytokines may contribute to tumor progression via autocrine/paracrine signaling, thereby exacerbating pancreatic cancer aggressiveness [101]. Hayashi M et al. further identified activation of the CXCL1–CXCR2 axis by *F. nucleatum*, which contributed to an immunosuppressive tumor microenvironment. Pharmacological inhibition of this axis suppressed tumor growth [68].

### Potential cooperative interactions

Microorganisms may contribute to pancreatic cancer initiation and progression through multiple mechanisms, and specific bacterial species may further promote tumorigenesis through cooperative interactions. As reviewed by Sun et al., the bacterial driver–passenger model provides a conceptual framework for understanding such interactions [88]. Within this model, *A. actinomycetemcomitans* may act as a driver by inducing host cell DNA damage and remodeling the microenvironment, thereby facilitating the proliferation and survival of additional pathogens; *F. nucleatum* may act as a passenger, localizing to cancer cells and serving as a bridging organism between early colonizers, including *A. actinomycetemcomitans,* and late colonizers, including *P. gingivalis*[88]. Meanwhile, *P. gingivalis* may inhibit cancer-cell apoptosis and promote tumor progression [88]. Metabolic heterogeneity among bacterial species may also support synergistic interactions. The proteolytic activity of *P. gingivalis* can supply amino acids to *F. nucleatum*, which lacks intrinsic proteolytic capacity, thereby facilitating bacterial co-colonization and forming a positive feedback loop that increases nutrient availability within polymicrobial communities. As summarized by Wang et al., *P. gingivalis* is mainly associated with immune evasion, apoptosis inhibition, EMT, and chronic inflammation, whereas *F. nucleatum* is associated with tumor localization, enhanced proliferation, immune suppression, and metastasis [102]. Together, these review-based observations raise the possibility that interactions between *P. gingivalis* and *F. nucleatum* may enhance tumor-promoting processes. Future research should clarify the molecular mechanisms underlying these interactions and evaluate whether polymicrobial signatures have potential as early diagnostic biomarkers and therapeutic targets in pancreatic cancer.

## Microbiota-modulating approaches and pancreatic cancer-related pathways

The proposed oral–gut–pancreatic axis has prompted interest in whether microbiota-modulating approaches can affect processes relevant to pancreatic cancer. This section discusses research on periodontal treatment, probiotics, and dietary interventions, particularly their effects on microbial communities and inflammation-related pathways.

### Microbiota-modulating approaches and inflammation-related findings

Periodontal therapy has been reported to improve oral dysbiosis and alter gut microbial composition [55]. Probiotics have also been examined in experimental models of pancreatic inflammation. For instance, Lutgendorff et al. reported that probiotic pretreatment significantly alleviated pancreatic injury in a rat model of acute pancreatitis [104]. The protective effects involved the suppression of NF-κB activation, the reduction of lipid peroxidation, and the enhancement of antioxidant capacity through increased pancreatic glutathione biosynthesis [104]. These findings suggest that probiotics may modulate inflammatory and oxidative stress responses in acute pancreatitis [104].

Moreover, experimental studies indicate that mice fed engineered resistant starch show altered gut microbial composition and reduced xenograft growth in a pancreatic cancer model [105]. In vitro, an engineered resistant-starch-mimicking medium reduced pancreatic cancer cell proliferation and ERK1/2 and mTOR phosphorylation [105].

### Microbiota and responses to pancreatic cancer treatment

#### Enhancement of immunotherapeutic efficacy

Accumulating evidence from animal models and clinical studies suggests that the gut microbiome may be associated with postoperative complications following pancreatic surgery and responses to immunotherapy [106]. In a 2021 study, researchers engineered an attenuated *Salmonella Typhimurium* strain to express cytolysin A (ClyA), targeting cancer stromal cells and tumor cells in mouse models of pancreatic cancer [107]. Assessment of the tumor microenvironment—including stromal cell abundance, marker expression, and immune cell infiltration—demonstrated that ClyA-secreting *S. Typhimurium* disrupted cancer stromal cells and enhanced immune cell infiltration, thereby mediating potent anti-cancer activity [107]. Furthermore, specific gut microbiota, such as *Megasphaera* species, have been associated with responses to PD-1 blockade [77,108].

#### Microbiota and chemotherapy responses

The influence of the gut microbiota on chemotherapy efficacy has garnered increasing attention. Specific gut bacterial species have been associated with altered chemotherapy responses, potentially through effects on host immunity and drug metabolism.

A 2023 study in *Nature* identified gut microbiota—including *Bacteroides fragilis* and *Bacteroides thetaiotaomicron*—that metabolize tryptophan into indole-3-acetic acid (3-IAA). Higher 3-IAA levels were observed in patients with pancreatic cancer who responded to chemotherapy. Experimental studies showed that 3-IAA enhanced chemotherapy activity by inducing ROS accumulation and reducing autophagy in pancreatic cancer cells [109]. In mice colonized with microbiota from chemotherapy responders, a short-term tryptophan-enriched diet increased serum 3-IAA levels and enhanced chemotherapy responses [109].

### Additional microbiota-related findings

Culture supernatants from *Bifidobacterium bifidum* and *Bifidobacterium breve* have been reported to inhibit EMT in pancreatic cancer cells in vitro [110]. This inhibition was accompanied by reduced Ki-67 expression and increased phosphorylated histone H2A.X (γH2AX) staining, indicating reduced proliferation and increased DNA damage, respectively [110]. These findings show that probiotic-derived products can affect tumor-related cellular processes in an experimental setting [110]. Fecal microbiota transplantation (FMT) has also been studied in mouse models to examine how donor microbiota relates to pancreatic cancer outcomes. Riquelme et al. transplanted fecal microbiota from patients with pancreatic cancer who had short-term or long-term survival, and from healthy controls, into mouse models [111]. After 35 days, tumors in mice receiving FMT from patients with short-term survival were significantly larger than those in the other two FMT-treated groups, suggesting that donor microbiota was associated with differences in tumor growth and antitumor immune responses in mice [111].

## Current limitations and future directions

Overall, epidemiological and experimental studies suggest a possible link between periodontitis and pancreatic cancer. However, several limitations complicate the interpretation of the current evidence. Most clinical studies are observational and may be affected by residual confounding from shared risk factors, including smoking, diabetes, obesity, diet, age, socioeconomic status, oral hygiene, medication use, and chronic inflammation. Microbial alterations detected after cancer diagnosis may represent either a contributing factor or a consequence of the disease. In addition, most studies have examined the oral, gut, and pancreatic microbiota separately, and direct evidence that oral microorganisms reach the human pancreas remains limited. The low microbial biomass of pancreatic tissue also increases the risk of contamination and makes microbial findings difficult to interpret. Furthermore, the diagnostic value of microbial markers and the effects of microbiota-targeted interventions have not been confirmed in large clinical studies.

Future studies should continue to explore the link between periodontitis and pancreatic cancer. Prospective longitudinal studies using standardized periodontal assessment, repeated oral and gut microbiome sampling, rigorous control of potential confounders, and strain-level microbial source tracking may help clarify the timing and possible origin of microbial alterations during pancreatic cancer development. Combining microbiome data with metabolomic and host gene expression analyses may also help explain the underlying mechanisms. In addition, patient-derived PDAC organoids could be used to examine the effects of selected microorganisms and their products. Potential microbial biomarkers should be validated in independent clinical cohorts, while microbiota-targeted interventions require further evaluation in clinical studies.

## Conclusion

This review examines the possible relationship between periodontitis and pancreatic cancer, with particular attention to microbial interactions along the proposed oral–gut–pancreatic axis and their potential underlying mechanisms. Periodontitis is closely linked to alterations in the oral microbiota, while pancreatic cancer development is associated with alterations in the pancreatic microbiota. Epidemiological studies suggest a possible association between periodontitis and pancreatic cancer, while experimental findings provide potential mechanistic explanations involving microbial interactions along the proposed oral–gut–pancreatic axis. Experimental studies in animal models suggest that oral microorganisms, including *P. gingivalis* and *F. nucleatum*, may reach the pancreas via the digestive tract or the circulatory system and interact with the local microenvironment. These microorganisms may exert pathogenic effects through their virulence factors and metabolic products. These activities may induce chronic inflammatory responses, facilitate immune evasion, and activate key oncogenic signaling pathways, including TLR/NF-κB and Wnt/β-catenin. These processes may be involved in PanIN progression and the development of pancreatic cancer.

Notably, the microbiota may serve not only as a potential contributor to carcinogenesis but also as a potential indicator of disease. Specific oral microbial features, such as increased abundance of *P. gingivalis*, and gut microbiota metabolic profiles, including reduced butyrate levels, have been investigated as candidate non-invasive biomarkers. Periodontal therapy, probiotics, and dietary interventions have been studied for their potential to alter microbial or inflammatory pathways relevant to pancreatic cancer; their effects on pancreatic cancer risk, progression, treatment response, and survival remain unclear.

## Data Availability

Data sharing is not applicable to this article as no data were created or analyzed in this study.
